# Determining the Needs, Priorities, and Desired Rehabilitation Outcomes of Young Adults Who Have Had a Stroke

**DOI:** 10.1155/2012/963978

**Published:** 2012-07-18

**Authors:** Maggie Lawrence, Sue Kinn

**Affiliations:** ^1^School of Health and Life Sciences, K413, Buchanan House, Glasgow Caledonian University, Glasgow G4 0BA, UK; ^2^Research and Evidence Division, Department for International Development, East Kilbride G75 8EA, UK

## Abstract

*Background*. Guidelines state that young adults' (aged 18–55 years) rehabilitation needs and priorities following stroke are different from older adults'. However, there is a lack of evidence regarding young adults' perspectives of their needs and priorities. *Aim*. To gain an understanding of young adults' experience of stroke and associated rehabilitation needs, priorities, and desired outcomes. *Methods*. A qualitative approach was adopted, based on the phenomenology of Merleau-Ponty. Longitudinal data were gathered using unstructured interviews and analysed using phenomenological reduction. *Results*. Ten young adults took part in up to four interviews over two years. An overarching theme, Embodied Disorientation, and three subthemes: Mortal Body, Situated Body, and Embodied Perception of Difference, described the young adults' experience. A subsequent iterative process enabled tabulation of patient-centred rehabilitation needs, priorities, and outcomes. *Conclusion*. Rehabilitation professionals can use the evidence-based outcomes table to work with young adults to develop meaningful patient-centred goals and select appropriate interventions which align with identified needs and outcomes throughout the stroke recovery trajectory.

## 1. Introduction

Stroke is a common, long-term condition, which is a principal cause of complex disability amongst those living in the community [1]. Stroke may affect physical, cognitive, and emotional functions and often causes major disruption to the life course [2]. It is often perceived to be a condition of older age, yet it is prevalent amongst younger adults, that is adults aged between 18 and 55 years [3]. Traditionally, the design and delivery of stroke services, both acute care and long-term rehabilitation, have reflected the needs and priorities of older adults. Increasingly, however, it is suggested that young adults' rehabilitation needs and priorities following stroke differ from those of older adults [4, 5], particularly in relation to parenting [6] and employment [7, 8]. An additional differentiating feature is that survival rates are high for young adults and, as a result, young adults are more likely to live longer with the physical, psychological, and social consequences of stroke [9].

Surveys conducted exclusively with young adults (aged 18–65), reported a range of unmet needs including personalised information about stroke, intellectual fulfilment, access to rehabilitation therapies, and opportunities for social reintegration [9, 10]. Qualitative studies conducted exclusively with young adults found that following stroke, young adults have a range of needs and desired outcomes, including those mentioned above as well as rehabilitation needs associated with sexuality, sexual function, employment, driving, self-confidence, and self-esteem [8, 11, 12]. Clearly, stroke has a significant impact on the lives of young adults and the body of research evidence relating solely to young adults is growing [5]. To date, however, the majority of this research has been conducted from the clinicians' perspective, and consequently, the focus tends to be on the first three to twelve months after stroke, the period when clinicians perceive that they are able to intervene most effectively in stroke care and rehabilitation [13]. Only a few studies have been reported which reflect the patient perspective (e.g., [14–16]), and a recent review of the qualitative literature [17] found only four qualitative studies conducted specifically from the perspective of young adults. This indicates a need for further research which enables young adults to provide accounts of their experiences of stroke and recovery from stroke over the longer term [18, 19] and thus provides the evidence required to inform development of patient-centred rehabilitation interventions. Therefore, we devised a programme of research that aligns with the development stage of the *MRC Framework for the Development and Evaluation of Complex Interventions* [20]. Our aim was to develop an evidence base which could be used to inform the design of tools and interventions to support patient-centred multidisciplinary rehabilitation practice in relation to the rehabilitation of young adults following stroke. The programme of research included a systematic review of patient-centred stroke literature [21] and qualitative interviews with young adults and with family members [22]. This evidence base informed our understanding of patient-centred rehabilitation needs, interventions, and desired outcomes. This paper provides a brief report of the qualitative interviews with young adults and detail of associated patient-centred rehabilitation outcomes, presented in tabular form. This patient-centred evidence may be used to assist rehabilitation professionals select appropriate interventions that align with the needs and priorities of young adults at different stages of the stroke recovery process. 

## 2. Aims

The qualitative study aimed to gain an understanding of young adults' experience of stroke over an extended period of time. A secondary aim was to use this new knowledge to inform our understanding of what constitute appropriate short-, medium-, and long-term patient-centred interventions and associated outcomes for young adults who have had a stroke.

## 3. Methods

Existential phenomenology was chosen as the methodological underpinning for this study [23]. A phenomenological inquiry endeavours to capture spontaneous accounts of a particular phenomenon, in this instance the experience of stroke, from a sufficient number of individuals to enable the researcher to identify points of convergence in the accounts and thus identify the essential structure of the phenomenon under investigation [24]. A longitudinal design was used to capture change in perceived needs and priorities over time.

A project Advisory Group was established which comprised young adults who had had stroke, family carers of young adults who have had a stroke and two health professionals who had experience of working specifically with young adults with neurological disorders. The group's contribution to aspects of the research process, including data analysis, reinforced the patient-centred nature of the research and its outcomes. 

## 4. Participants

It is notable that definitions of “young” vary considerably between research studies for example 18–49 years (e.g., [25]) and 18–65 years (e.g., [5, 9]). In this study, we adopted the definition 18–55 as used by Corr and Wilmer [8] in their qualitative study of employment issues after stroke.

We recruited young adults from four National Health Service Health Boards via clinical “gatekeepers,” that is, three stroke nurse specialists and one consultant physician. Inclusion criteria specified that potential participants should be aged between 18 and 55, have had a stroke at least three months and up to two years previously, and be willing to talk to a researcher about their experience of stroke. Participants were required to be at least three months after stroke to ensure that they were out of the acute phase of recovery and were up to two years after stroke to reflect the variable nature of the stroke recovery trajectory (e.g., [2, 12, 25]). We used purposive sampling methods. Variables in our sampling frame were marital status, family structure (e.g., children/no children), location (i.e., urban/rural), and prestroke employment status.

## 5. Data Collection

Interviews were conducted in participants' homes or other location of their choosing. Participants were asked a single opening question, that is, “Please tell me about your experience of stroke;” no further questions were posed. In the ensuing interview, the researcher M. Lawrence adopted a “phenomenological stance” that used active and supportive listening skills to support the spontaneous speech of the participant [24]. This method allows participants to talk about topics that are important to them, rather than addressing the researcher's agenda [24]. Interviews lasted between 15 minutes and one hour and were conducted at six-month intervals over the course of two-years. Six-month intervals were considered sufficient to observe any change between interview periods. Data were collected over a two-year period to facilitate exploration of change over time, within the constraints of a time-limited study. In subsequent interviews, participants were not directed to reflect on previous interviews nor were they asked to reflect on the period that had elapsed between interviews; rather, participants were supported to speak of what was important to them at that time.

## 6. Data Analysis

All interviews, except one which failed to record due to operator error, were digitally recorded and transcribed. Analysis was conducted using a three-stage, iterative process: description, reduction, and interpretation [24]. In stage one (phenomenological description), a phenomenological stance was adopted, as described above, which enabled the researcher to obtain participants' prereflexive accounts. During the second stage, phenomenological reduction, the researcher's knowledge and experience were brought to bear on the data and in a process of critical reflection, the researcher endeavoured to “see” the phenomenon from all perspectives. This required immersion in the data, which was achieved by listening repeatedly to the interview recordings, often whilst reading the transcripts. The Advisory Group played an important role in this stage of the analysis, as both their spontaneous and considered responses to the anonymised interview excerpts provided yet another perspective on the data.

By means of these processes, meaning units (a discrete segment of a transcript that can stand alone as a single idea) were identified and, in a further analytic iteration, themes were identified from the convergence of meaning units. Stage 3, phenomenological interpretation, involved an iterative process comprising alternating periods of reflection, writing, and modeling. During this stage, M. Lawrence held a team analysis meeting. The team comprised three colleagues (see Acknowledgments), all experienced in qualitative data analysis in stroke or older adult research. The purpose of the meeting was to try to understand how the previously identified themes related to one another, to identify overarching themes, and to develop third-order constructs which represent a descriptive account of the experience or phenomenon under investigation [24, 26] (see Supplementary file 1 available online at doi:10.1155/2012/963978). By the end of the analysis meeting, we had agreed on a thematic model which, in subsequent iterations of analysis, M. Lawrence developed further, using the modeling feature of NVIVO, a qualitative data analysis software package (supplementary file 2). 

## 7. Rigour 

Meyrick [27] described two key principles of rigorous qualitative research: transparency and systematicity. Phenomenological writing is a controversial act due to the unique perspective of each researcher [28]. This inherent “uniqueness” reinforces the need for transparency of reporting. For example, decisions made throughout the research process should be documented and made available for scrutiny. Systematicity requires that the researcher should consistently apply congruent methods of data collection and analysis throughout the research process, and any deviations from the stated design should be described and justified [27]. Systematicity and transparency were the criteria adopted in this study as the means of ensuring its quality and rigour [29]. 

## 8. Ethics

The study was approved by the university Ethics Committee and the National Health Service Central Office for Research Committees. 

## 9. Results

The study participants included five men and five women; demographic details are provided in Table 1. Over the course of two years, they participated in a maximum of four interviews. Five took part in four interviews, two took part in three interviews, and three took part in two interviews. Reasons for attrition included family bereavement and work-related stress.

Following the analytic process described above, the experience of stroke from the perspective of young adults was described as Embodied Disorientation. Three subthemes were identified: Mortal Body, Situated Body, and Embodied Perception of Difference.

## 10. Embodied Disorientation 

In phenomenology, the body is understood to be the medium through which individuals perceive and understand the world and their place within it. Stroke impacted suddenly and dramatically; the young adults found themselves suddenly disoriented. They experienced their body ambiguously; it was both familiar and unfamiliar [23, page 95]. This disorientation provoked uncertainty and fear:

**It hit me very hard. For three or four days, I was just hysterical … I was so unsure … all I wanted to know was if I could walk or not, and all they could tell you was, “You'll have to wait and see.” … so many other questions I asked the staff, they couldn't answer. (Andrew, T1)**

For many participants, in spite of apparently making a good recovery from affective, physical, and cognitive effects of stroke, this perception of being disoriented persisted over time. For example, most participants who had been employed at the time of their stroke recovered well and regained a sense of embodied orientation. However, when they returned to work they found that the demands of the workplace highlighted cognitive and other poststroke impairments that had not been noticeable in their familiar home environment. This reawakened a sense of embodied disorientation. They experienced a tension between their familiar sense of self and their embodied self following stroke. This overarching theme of embodied disorientation formed the background to the young adults' experience of stroke. 

## 11. Mortal Body 

The suddenness of stroke and its devastating effects caused the young adults to become suddenly aware of and to reflect upon their own mortality. Prior to stroke, the young adults took for granted their ability to participate in everyday activities. However, with the onset of stroke, they were shocked out of this taken-for-granted way of being:

**I found the worst thing is … the devastation it caused just in seconds like that … you think, “Is this it?” (Adam, T1)**

Often, stroke had hit without warning; it came “out of the blue” and young adults lived with the fear that it would happen again:

**Every time I don't feel a hundred percent I think, “Oh no! Is this me taking something major?” (Lorraine, T1)**

## 12. Situated Body 

Following the disruption caused by stroke, young adults' sense of being orientated in a familiar world was destroyed in an instant. This caused them to reassess the ways in which they participated in the world and how they interacted with others. They struggled to reorient themselves and regain their familiar understanding of themselves in the world. They reviewed their lives, their priorities, and their life approach. Often, their attitude to life changed to accommodate new insights and understandings. They used a range of psychological coping strategies, such as choosing to adopt a positive mental attitude:

** (At first you are depressed) then (you) get a grip of yourself and you think, “Right, come on! I've got to try something here. I've got to look for positives!” (Andrew, T1)**

Once the immediate crisis had passed, the young adults' main aim was to regain prestroke “normality.” Often, they described themselves as being unaffected by stroke and ready to engage with the world as they had done before their stroke. However, the claim *“stroke has not affected me,”* made by most participants, was either preceded or succeeded by a description of the ways in which stroke had negatively affected life and prevented them from resuming normality:

**Stroke (has) not stopped me in anyway, you know, it's not made my life any different apart from … I don't like going to (town) or anything without a friend now … I used to be able to (keep going until) two in the morning … not now (Audrey, T1)**

## 13. Embodied Perception of Difference

Following stroke, young adults perceived that their bodies were different and consequently the ways in which they perceived and understood themselves as an individual with a familiar role and place in the world had changed:

**There's certainly a change in my attitude, a change in my personality, a change in everything really. (Gordon, T3)**

Generally, when participants spoke about a perceived difference in their sense of self, it was in relation to responding to stressors and to other people differently, or feeling that they would never be entirely the person they had been prior to stroke:

**I would say I'm 95% back to what I was. I don't think you ever get the other 5% (Cathy, T1)**

However, most participants did not overtly refer to an altered sense of self. In fact, they often stated the opposite: *“I don't feel any different”* and *“My friends say I'm not the same person; I think I am.” *Ambiguously, however, the follow-on from such statements was a description of at least one aspect of life that had changed following stroke. Only one young adult described his perception of personal difference in positive terms. Andrew felt that he was a better person as a result of his experiences following stroke.

Fatigue was described by most participants. It affected memory and concentration. Participants who had returned to work found that they were exhausted; they had no energy, and were unable to participate in other activities. Tiredness also affected family relationships. Participants described themselves as being argumentative, lacking in patience, and being more emotionally labile following stroke. They became uncharacteristically upset and experienced angry outbursts, often sparked by minor irritants.

Participants noticed many different cognitive effects of stroke, including memory problems, poor concentration, visual impairments, and impaired decision-making skills:

**I find that I get tired quite easily, and I put off making decisions about things, because I can't be bothered (laughs). (David, T3)**

The young adults also described an altered sense of self which affected their perception of how they were perceived by and related to other people. It included aspects of body image, self-image, and self-esteem; complex issues about which several young adults had difficulty articulating their feelings. This was due in part, perhaps, to a lack of understanding of their ambiguous responses to these issues. For example, one young adult who had left-sided hemiplegia described being *“embarrassed by my condition.”* His emotional response to his altered, physical, circumstances was complex and ambiguous. He described the dissonance between the way he thought about himself and the reality:

**I see my sons go out and play football (and) I want to go out and play with them—I can't! It's not even remotely possible for me to do it … but I still want to do it … the fact is I just can't do it any more at all, but you really don't think that way, your brain still says to you, “I can do that!” (Gordon, T4)**

Some participants described an awareness of changes in their physical appearance and differences in physical and cognitive function which caused them to worry about the ways in which others, particularly strangers, might respond to them. These worries and fears were such that an individual's participation in family, work, and social life was restricted when compared with life before their stroke. Busy public places were perceived as particularly difficult; crowds were experienced as intimidating and hard to negotiate, particularly if the young adult's mobility was impaired. For some, the dislike or avoidance of crowds and crowded places, or of being in public was associated with a disturbance of their sense of self, associated with negative changes in self-esteem and self-image, resulting from altered physical and cognitive function. For example, one young adult described panicking when she first visited a supermarket after her stroke; she anticipated that her now unfamiliar body would let her down:

**I thought, “I don't have any money, I only have my (bank) card and I'll need to write my signature,” and I started panicking … I panicked more than normal, I've lost all my confidence … I (was) quite a confident person (before my stroke). (Cathy, T1)**

## 14. Identification of Patient-Centred Needs ****and Associated Rehabilitation Interventions ****and Outcomes

As described above, an iterative process of phenomenological analysis was used to develop a framework to describe the experience of stroke from the perspective of young adults. Subsequently, using this framework and associated findings, M. Lawrence engaged in a further round of iterative reflective analysis which enabled extrapolation of patient-centred needs and subsequently, identification of associated patient-centred outcomes (Table 2). The identified needs and outcomes presented in Table 2 were validated in discussions with the Advisory Group [29]. Using the qualitative findings as the basis for this process ensured that the patient-centred needs and outcomes articulated in Table 2 are firmly based in research-generated evidence [30]. 

## 15. Discussion

This paper reports the findings from a qualitative investigation of the experience of stroke from the perspective of young adults who have had a stroke. The design and conduct of the research was congruent with major precepts of Merleau-Ponty's existential phenomenology [23]. Phenomenological analysis revealed that the experience was characterised by an overarching sense of disorientation, in which three subthemes were described: Mortal Body, Situated Body, and Embodied Perception of Difference. Similar themes were noted by Immenschuh [12] in her study of young adults and have also been noted in studies of general stroke populations (e.g., [31]). Controversially, this suggests that these experiences are universal following stroke and not specific to young adults as has been suggested elsewhere in the literature and in clinical guidelines (e.g., [4, 8–10, 25]). Young adults' needs and associated rehabilitation outcomes in the short-, medium-, and long-term following stroke were extrapolated from the qualitative findings and confirmed by the project Advisory Group (Table 2). Over recent years it has been suggested that stroke rehabilitation professionals should provide interventions designed especially for young adults (e.g., [4, 25]), but the findings from this study suggest that young adults' needs and priorities, and associated rehabilitation goals, are similar to those of the general stroke population. However, systematic review evidence revealed that patients want to be involved in setting goal, but that in practice still tends to be clinician-centred or even system-centred [32]. We argue that age-specific interventions are not required. Rather, stroke rehabilitation professionals should conduct an holistic assessment of individual's needs and priorities to ensure that these are identified and addressed, relevant rehabilitation goals are set, and interventions are implemented that are perceived to be appropriate and meaningful to the individual, irrespective of age [21, 29]. 

Being a young adult who has had a stroke was experienced in terms of bodily disorientation. They experienced their bodies ambiguously as familiar and unfamiliar; they felt the same, yet different. After the initial shock of the stroke event, young adults were aware of their own mortality. They realised that life could not be taken-for-granted and were afraid of an uncertain and unknowable future [33]. These experiences have been described in other studies of young adults (e.g., [12]) and in studies of the general stroke population (e.g., [31]). Rehabilitation professionals need to be alert to and acknowledge this awareness of mortality and address some of the associated issues, fear of dying, fear disability, by listening to young adults' fears and worries [34], and by providing appropriate psychological interventions [25]. Clinical guidelines suggest that all members of the multidisciplinary team should be able to identify and manage psychological issues, even if their role is simply to recognise problems and refer to, as appropriate [35, 36].

Young adults feared recurrence of stroke, and this fear endured over time [33]. However, some participants found it difficult to make changes to lifestyle behaviours, such as smoking, eating unhealthy, and being physically inactive, that put them at risk of recurrent stroke [37]. Evidence-based guidelines recommend implementation of multimodal secondary prevention interventions, which incorporate prescription of medication, information, and education [38, 39]. Such interventions should be theoretically informed and tailored to the needs and priorities of stroke survivors and their families [39, 40].

The experience of stroke caused the young adults' to reflect on their attitude to life and their priorities, a theme frequently reported in qualitative stroke research (e.g., [17, 41]). Ambiguously, however, the young adults also sought to return to normal, that is, to life as they knew it before stroke. For many, employment was an important aspect of prestroke normality, and they sought to return to employment as soon as possible, using this as a marker of successful rehabilitation and recovery. Often, however, they struggled; fatigue and cognitive impairments were considerable barriers, and they lacked the information and support they felt they needed [8, 42]. Corr and Wilmer [8], who investigated young adults' experiences of employment after stroke recommend implementation of comprehensive, well sign-posted, vocational programmes, delivered in partnership with employers and other agencies. And, importantly, it is essential that rehabilitation professionals ascertain the young adults' expectations and work with them to attain mutually agreed achievable goals [21, 32]. 

Following stroke, the young adults felt different although, ambiguously, they persisted in denying being different. Often, public spaces and crowds were perceived as difficult and intimidating. They felt that others perceived them and reacted to them differently than prior to their stroke. Relationships with other people, including family members, were altered. These findings reflect findings from studies of young adults and studies of general stroke populations (e.g., [25, 31, 33–44]). Clinical guidelines recommend that rehabilitation professionals implement psychological interventions that promote effective use of coping skills and other mechanisms to support adaption (e.g., [4, 35]). However, family functioning should also be assessed, particularly since family support is thought to be effective in improving rehabilitation outcomes [22, 45].

### 15.1. Limitations

The small study sample (*n* = 10) represents a potential limitation of this study. However, small numbers are common in phenomenological studies (e.g., [31, 44]); Thomas and Pollio [46] suggest that between six and twelve is appropriate. Another potential limitation relates to lack of heterogeneity in the sample, specifically with regards ethnicity and stroke severity. As recruiting health board areas included cities in their catchment, it was expected that the sample would include participants from different ethnic groups; however all potential participants referred to the researcher were Caucasian. In terms of stroke severity, only one a young adult had had a “severe” stroke resulting in severely limited functional ability. The majority of young adult participants had made a good recovery that is they had few “visible” effects of stroke [47]. However, as one of the differentiating features of stroke in young adults is rapid physical recovery [48], it is likely that participants were representative of a young adult stroke population. 

## 16. Conclusion 

The qualitative findings from this phenomenological study enabled a deeper understanding of the experience of stroke from the perspective of young adults, from which short-, medium-, and long-term rehabilitation needs, priorities, and associated outcomes were identified. Drawing on this understanding and on available research evidence, and combined with professional experience and expertise, rehabilitation professionals may use the table of patient-centred needs and outcomes to identify short-, medium-, and long-term rehabilitation needs and priorities of young adults, and work with young adults to develop meaningful goals which are of mutual benefit to patients and families. Appropriate interventions may then be selected which align with identified patient-centred needs and outcomes throughout the stroke recovery trajectory. 

## Supplementary Material

Supplementary file 1: Outputs of the team analysis meeting. This file contains sample digital photographs taken during the team analysis meeting. The photographs provide an example of ‘work in progress' during the development of an overarching theme based on the existential concept of Body.
Supplementary file 2: An example of concept modelling using NVIVO software: Body. This file is a screen shot from NVIVO showing how the results of the team meeting were ‘translated' into the conceptual mapping feature of NVIVO for further analysis.

## Figures and Tables

**Table 1:** Participants' demographic details. Key: p/t: part-time; f/t: full-time; s/e: self-employed.

Name	Age	Marital status	Children	Rural/urban	Employment status before stroke	Months after stroke
Adam	48	Married	1 at primary school	Village	f/t site agent (construction industry)	9
Andrew	39	Single	None	Town	Long-term sick	5
Audrey	46	Divorced	1 daughter (aged 18) lives at home; 2 others married with children	Town	p/t voluntary work and “childminder” for grandchildren	5
Cathy	44	Separated	1 at secondary school; 1 aged 20; both at home	Town	f/t call centre operator	9
David	50	Divorced; remarried	2 children at university; both live with mother	Village	s/e retail manager	14
Gordon	43	Married	2 at secondary school	City	Long-term sick	22
Jim	45	Divorced	1 at secondary school; 1 doing apprenticeship; both live with mother	City	Long-term sick	7
Juliet	48	Divorced	1 daughter taking a “year out”	City	f/t human resources manager	5
Lorraine	37	Married	2 at primary school; 2 at secondary school	Town	p/t nurse	3
Norma	54	Married	2 adult children	Town	p/t machinist	5

**Table 2:** Patient-centred needs and outcomes. Key: I: information; E: education; M: medical needs; PS: psychological support; R: rehabilitation; PA: practical assistance.

Short-term effects	Short-term needs/rehabilitation interventions	Patient-centred rehabilitation outcomes

Mortal body		
Shock	Psychological support (PS)	Accommodation of the event to the individual's altered way of being
Fear of recurrence	Diagnosis and treatment of cause (M; I) Explanation of treatment and cause (M; I) Information provision (I) Psychological support (PS) Secondary prevention (M; I; E)	Understanding of cause and treatment Fear is manageable and does not hamper daily living Secondary prevention strategies are in place Nonrecurrence

Intermediate effects	Intermediate needs	Patient-centred outcomes

Situated body		

Attitude to life Coping	Education: effects of stroke and likely outcomes (I; E) Coping skills: assessment and training (PS)	Knowledge of effects of stroke and likely outcomes Implementation of effective coping skills and strategies
Reclaiming normality Stroke has/has not affected me Work	Psychological support (PS) Management of fatigue (PS; R) Rehabilitation: individual's role in the family (R) Rehabilitation: social/leisure activities; vocational rehabilitation; driving (R) Identification of practical support needs (PA)	Awareness and acceptance of impact of stroke on personal identity Resumption of former roles and/or Adaptation to altered ability to participate in former roles

Long-term effects	Long-term needs	Patient-centred outcomes

Embodied perception of difference		

Sense of difference Tiredness Cognitive effects	Psychological effects of stroke: diagnosis, assessment and education (PS) Management of fatigue (PS; R) Cognitive effects of stroke: diagnosis, assessment and education (R; PS)	Effective management of psychological effects of stroke Effective management of fatigue Effective management cognitive impairments
Social difference Relationships Being in public	Body image, self-esteem: assessment (PS) Sense of self: awareness and understanding (PS) Self-efficacy (I; PS) Practical support: assessment and equity of access (PA) Family function: assessment; relationship counselling (PS)	Awareness and acceptance of impact of stroke on body image, self-esteem, and sense of identity/self Resumption of former relationships (family and society) Acceptance of sameness/difference Effective family functioning; “healthy” relationships

The table is divided into three sections: mortal body, situated body, and embodied perception of difference, which reflect the subthemes from the qualitative interviews. These sections are mapped to short-, medium-, and long-term effects of stroke. This temporal perspective reflects the ways in which the experience of stroke and associated needs and outcomes change over time. The table comprises three columns, that is, effects of stroke, patient-centred needs/rehabilitation interventions, and patient-centred rehabilitation outcomes.
