# Supplementary figures and images for: Determining the Needs, Priorities, and Desired Rehabilitation Outcomes of Young Adults Who Have Had a Stroke
Source: Rehabil Res Pract. 2012 Jul 18;2012:963978. doi: 10.1155/2012/963978 (PMC3407657; doi:10.1155/2012/963978)

Supplementary file 1

Outputs of the team analysis meeting


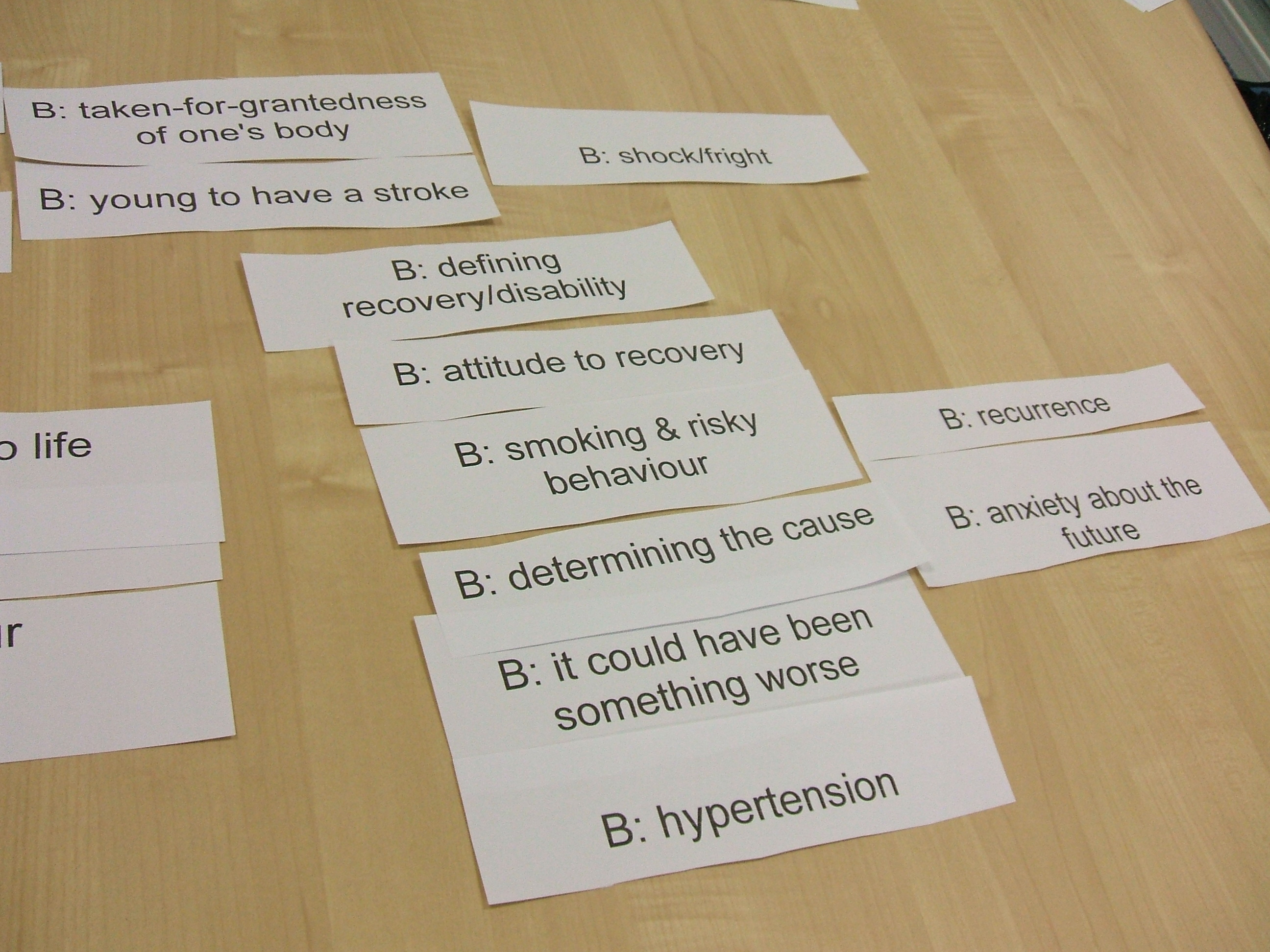


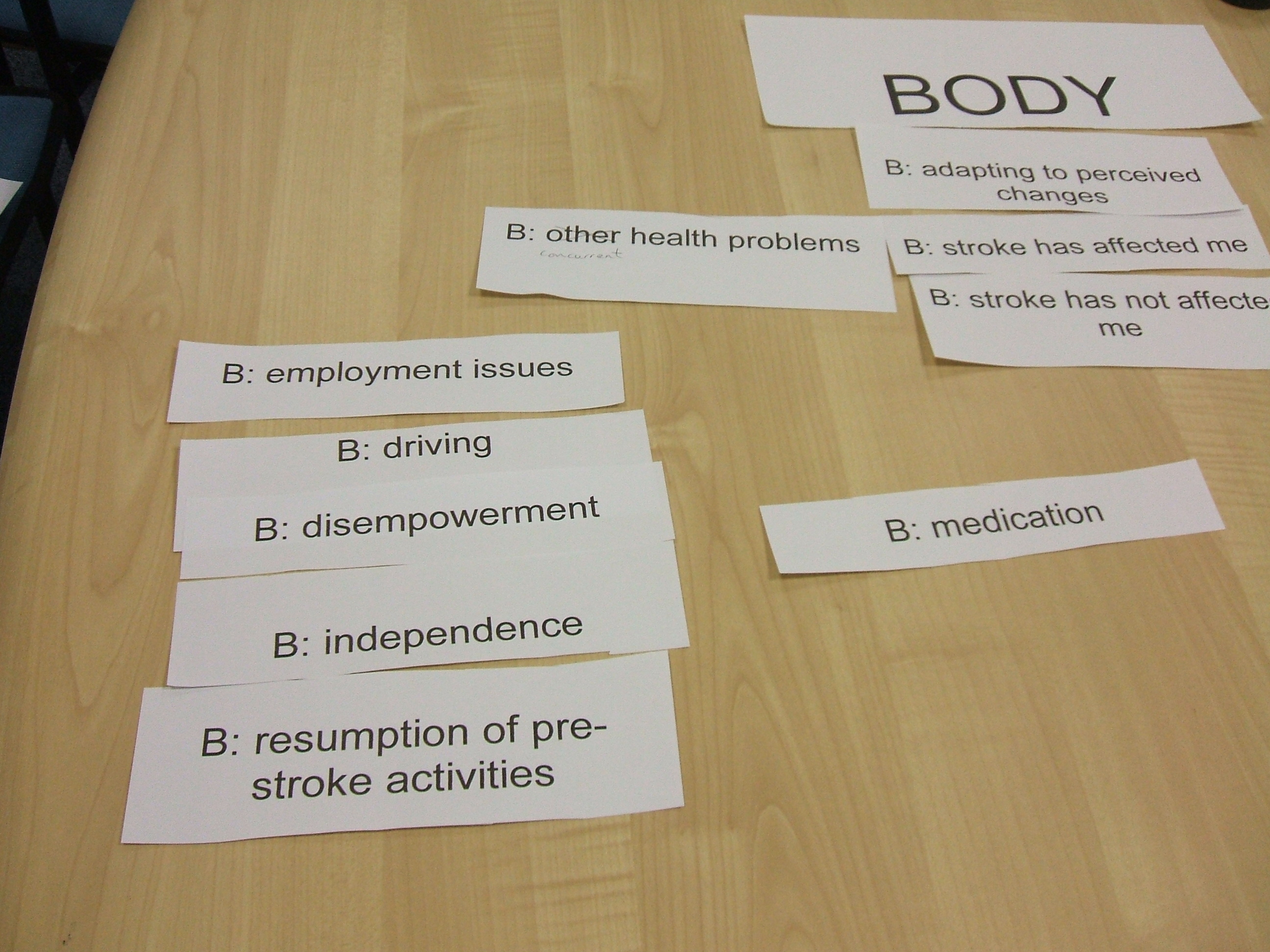

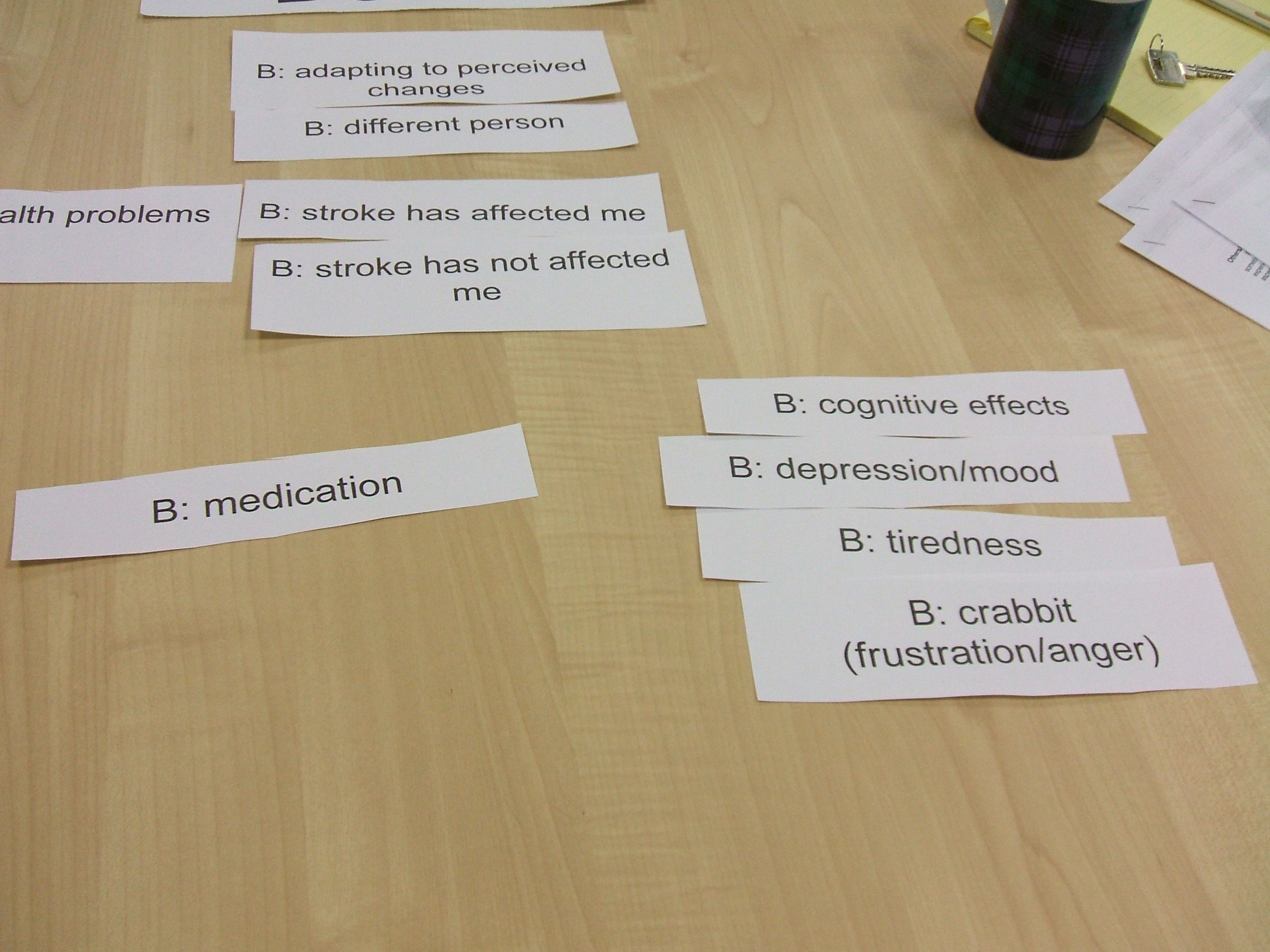

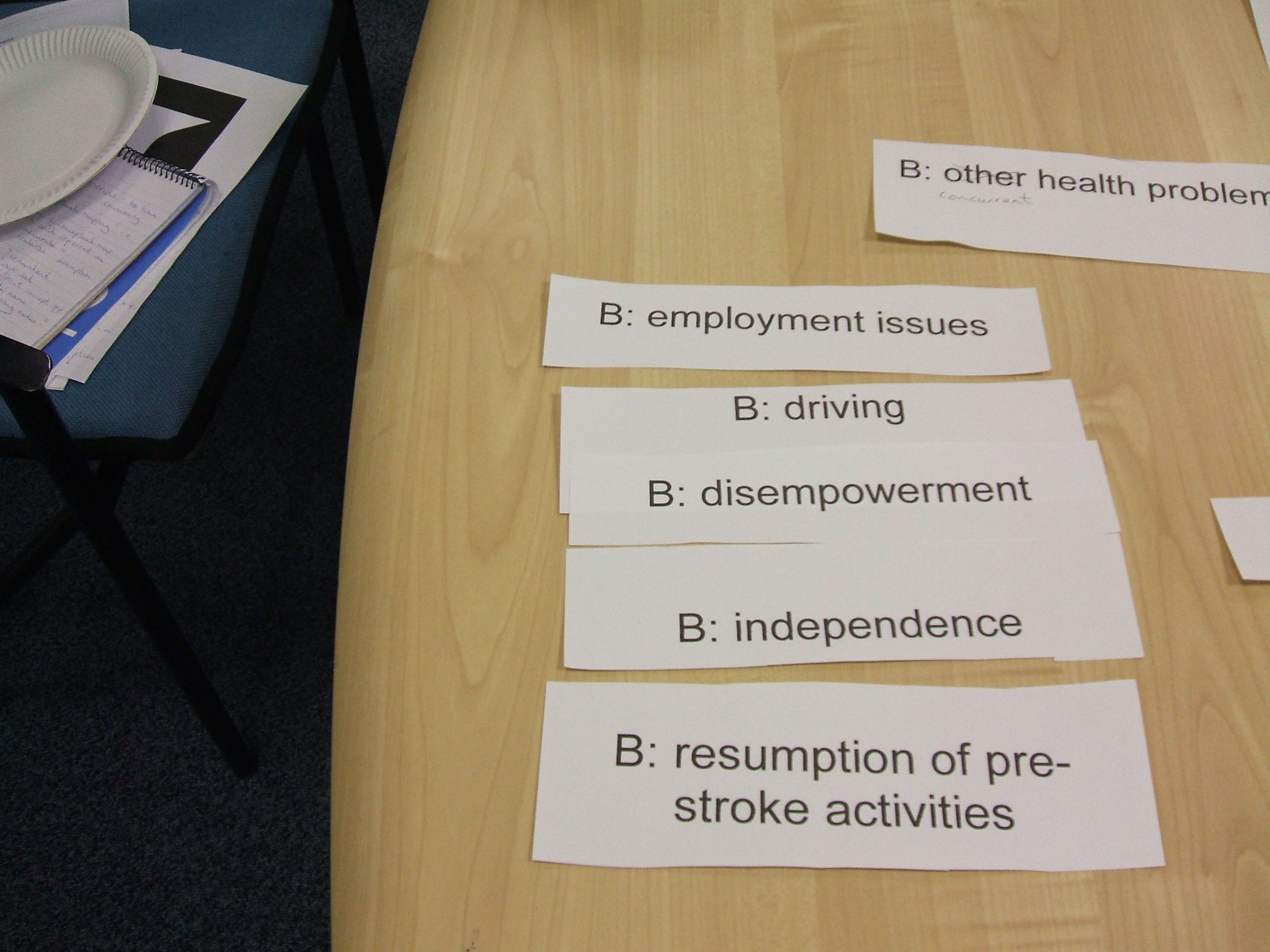

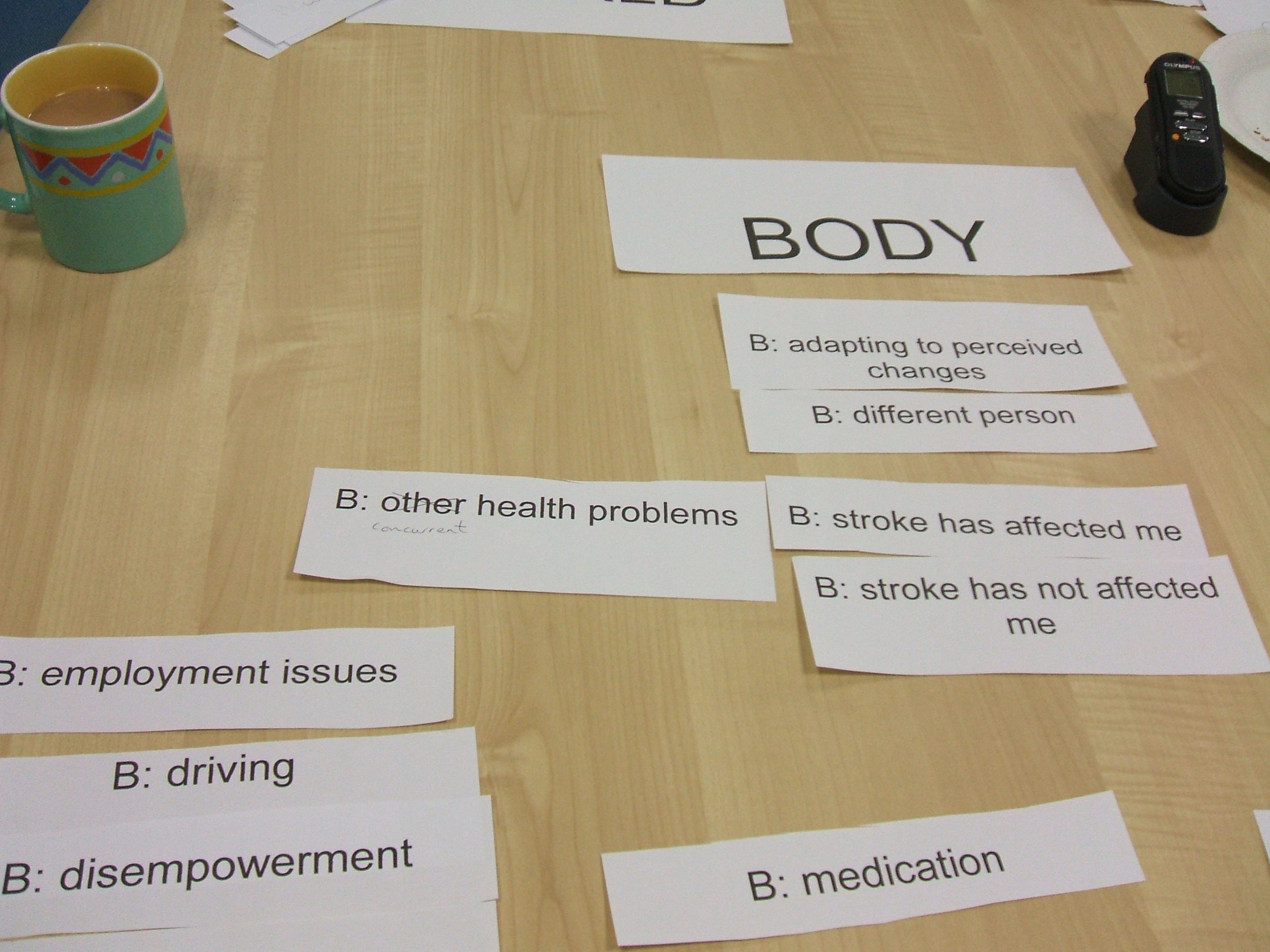

Supplement: Supplementary file 1 — Outputs of the team analysis meeting. This file contains sample digital photographs taken during the team analysis meeting. The photographs provide an example of ‘work in progress' during the development of an overarching theme based on the existential concept of Body. Supplementary file 2: An example of concept modelling using NVIVO software: Body. This file is a screen shot from NVIVO showing how the results of the team meeting were ‘translated' into the conceptual mapping feature of NVIVO for further analysis. [file 963978.f1.doc]

Supplementary file 2

An example of concept modelling using NVIVO software: Body


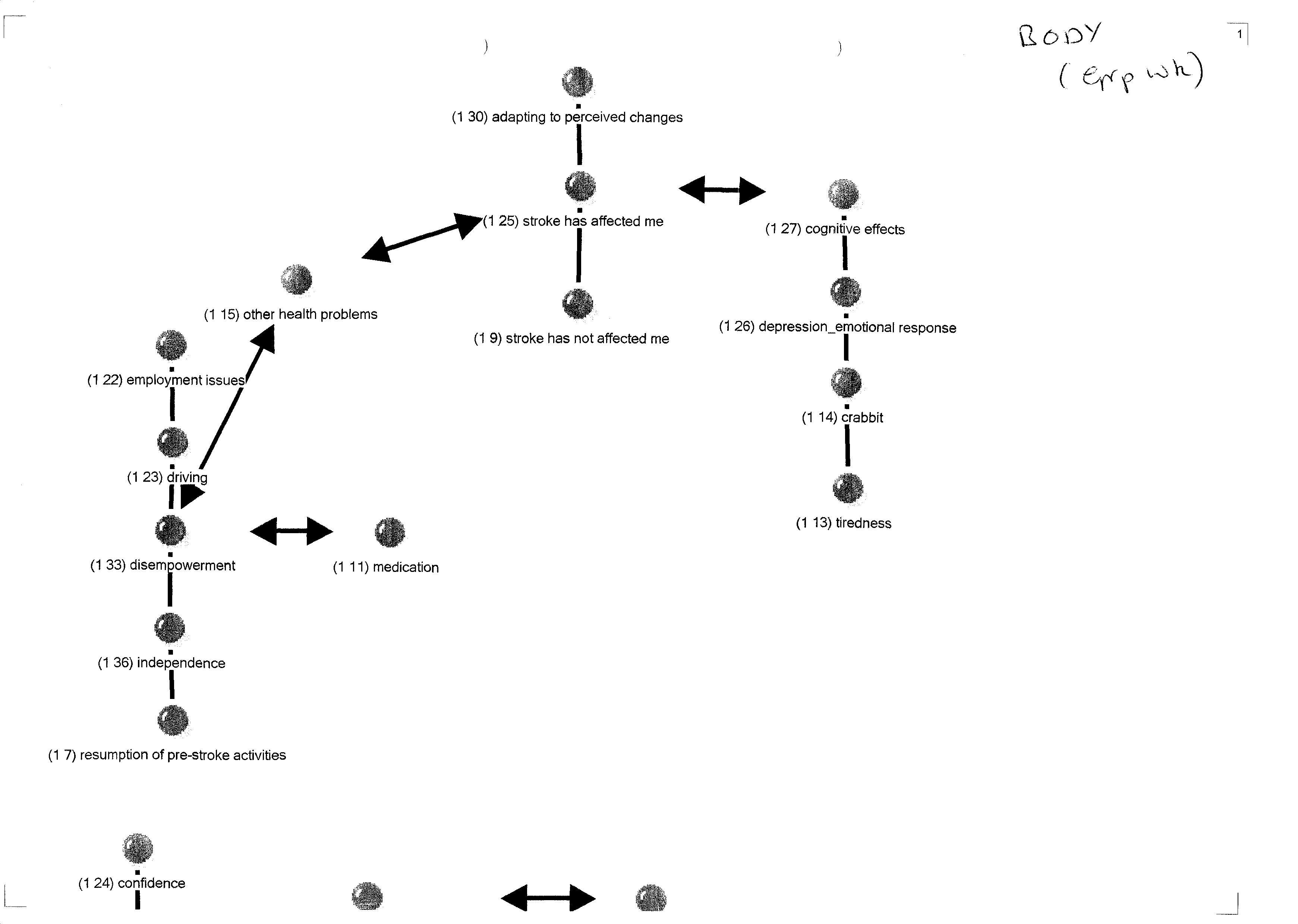

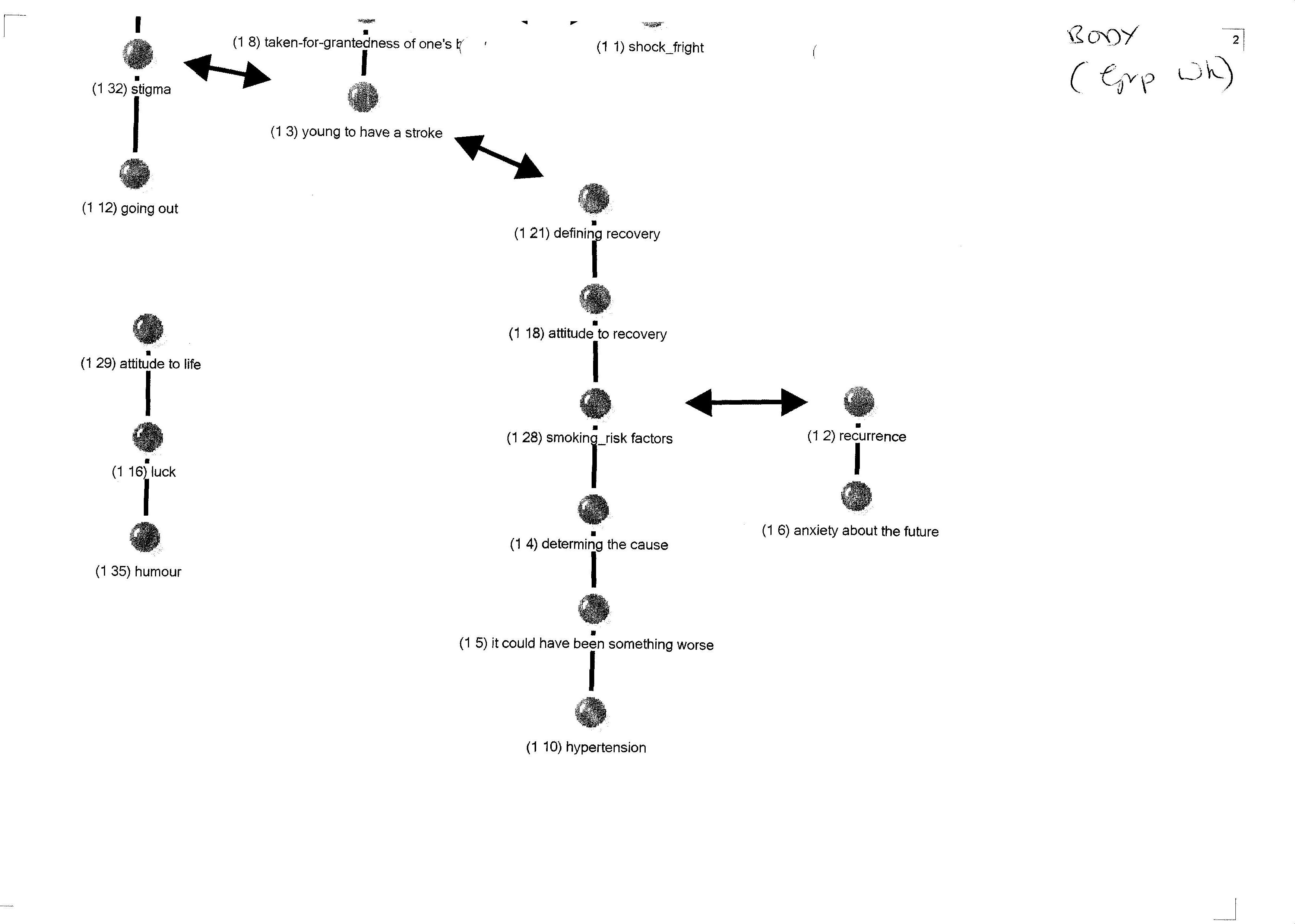

Supplement: Supplementary file 2 [file 963978.f2.doc]
